# Flipping the sex switch: Genetic insights into sex determination factor in *Ceratopteris richardii*

**DOI:** 10.1093/plcell/koaf079

**Published:** 2025-04-02

**Authors:** Sonhita Chakraborty

**Affiliations:** Assistant Features Editor, The Plant Cell, American Society of Plant Biologists

When it comes to reproduction and sex determination, the seemingly innocent fern harbors a deep secret. Unlike flowering plants, where sex determination is linked to chromosomes, homosporous species produce spores. The spores produced by diploid sporophytes have the potential to develop into gametophytes that are either haploid males or haploid hermaphrodites (Figure A). External signaling molecules and environmental cues play an important role in determining the sex of these gametophytes. When spores of the homosporous fern *Ceratopteris richardii* are exposed to the pheromone antheridiogen of *Ceratopteris* (A_CE_), secreted by hermaphroditic fern gametophytes, they develop into male gametophytes (Hornych et al. 2021). The identity and origins of A_CE_ and how it affects sex determination have remained a mystery. In new work, **Katelin M. Burow and colleagues** (Burow et al. 2025) begin to unravel the mysteries of this sexual determination saga by identifying the pivotal player required for A_CE_ to promote male gametophyte development as an ortholog of the well-known receptor-like kinase BRASSINOSTEROID INSENSISTIVE 1 (BRI1).

**Figure.**
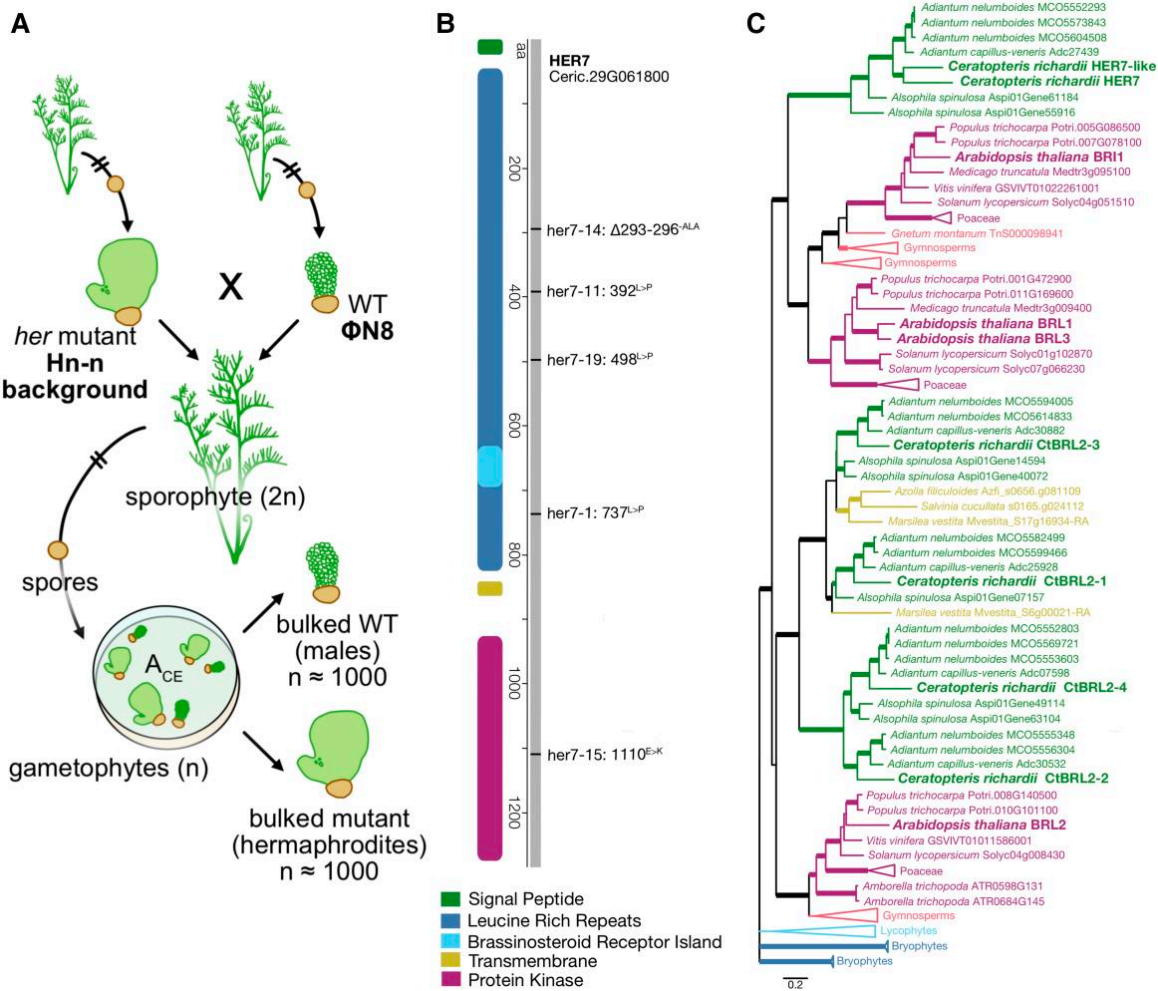
An overview of *Ceratopteris richardii*. **A)** The life cycle of *C. richardii* comprises the diploid (2n) sporophyte giving rise to spores, which in turn may develop into haploid (*n*) gametophytic hermaphordites or males. **B)** The location of the 5 independent *her7* mutations at the HER7 locus. **C)** The GeneRax-optimized maximum likelihood phylogeny of the BRL gene family in plants. Color codes represent homosporous ferns (green), heterosporous ferns (yellow), angiosperms (purple), gymnosperms (pink), lycophytes (light blue), and bryophytes (dark blue). Arabidopsis and Ceratopteris homologs are bolded. Adapted from Burow et al. (2025), Figures 1, 2, 3.

A major advantage to studying ferns over seed plants is the ease with which fern spores may be mutagenized to produce a plethora of mutants that can quickly be sorted based on the highly dimorphic sexual identity of the gametophytes (smaller male versus larger hermaphrodite). The hermaphroditic (*her*) linked mutants are one such group of mutants that were identified and selected due to their insensitivity to A_CE_ and inability to develop as hermaphrodites despite exposure to the antheridiogen (Warne et al. 1988). The large genome of *Ceratopteris* has made it challenging to identify the causative genes of these mutations. Thanks to the recent full genome sequencing of the *Ceratopteris* genome (Marchant et al. 2022), the authors were able to perform bulked segregant analysis RNA-seq on an F2 population segregating for the mutation and map the *her* mutations to the Ceric.29G061800 locus (dubbed HER7) encoding a putative receptor-like kinase (RLK). The various *her* mutations were found to occur in the leucine-rich region and kinase domain of the putative RLK (Figure B). Transient expression of *35S::GFP-HER7* in *N. benthamiana* leaf epidermal cells confirmed its predicted localization to the plasma membrane, like that of other RLKs. The accumulation of HER7 transcripts in gametophytes treated with A_CE_ further suggested that the putative RLK HER7 is required for A_CE_ to exert its effect on sexual determination in *C. richardii* (Burow et al. 2025).

Burow et al. (2025) further investigated the evolutionary relationship of BRI1-like receptors (BRLs) within the broader context of land plant brassinosteroid (BR) receptors. Phylogenetic analysis using sequences of BR receptors from various plant lineages revealed that a homosporous fern-specific clade (comprising HER7 and the paralog HER7-like) clusters close to its seed plant-specific sister clade (comprising Arabidopsis BRI1 and other land plant BR receptors) (Figure C). Although other *Ceratopteris* BLRs were found to group with homosporous and heterosporous ferns, the distinct clustering of the HER7-containing clade and its absence in heterosporous species hints at the possible existence of fern-specific lineage of BR receptors that needs to be further characterized to shed light on the evolution of hormone signaling. Although ferns are known to have BR metabolites, we are only just starting to understand them and their effects on fern physiology (Yokota et al. 2017). This prompted the authors to explore how genes involved in BR signaling and biosynthesis might be differentially expressed between males and hermaphrodites. Burow et al. (2025) observed an upregulation of BR biosynthetic enzymes (including CYP90) in hermaphrodites. However, genes involved in gibberellin signaling and biosynthesis, previously shown to play a role during sex determination in *Ceratopteris*, did not show obvious trends. The authors wondered whether A_CE_ functioned as a BR for its receptor, HER7.

With this work, Burow and colleagues (2025) open many exciting research questions: How does HER7 function as a receptor kinase in the sex determination pathway? What are the molecular mechanisms by which A_CE_ influences undifferentiated gametophytes to develop as males? By uncovering the genetic basis of sex determination in the homosporous *C. richardii*, this study provides a comparative framework for studying the evolution of sex determination across diverse plant species.

## Recent related articles in *The Plant Cell*

- Mabry et al. (2024) discuss the advantages of developing genomic resources for entire plant phylogenetic orders, leveraging trait diversity to enhance our understanding of plant biology.
- Charlesworth and Harkess (2024) highlight the importance of plant sex chromosome research and its indispensable role in understanding the complex relationships between developmental genetics, genome evolution, and evolutionary ecology.
- Hu et al. (2023) show that the BIN2-VLG signaling module regulates the timing and formation of the large vacuole, which is essential for proper female gametophyte development in *Arabidopsis.*

## Data Availability

No new data were generated or analysed in support of this research.

## Dive Curated Terms

The following phenotypic, genotypic, and functional terms are of significance to the work described in this paper:

BRI1 Gramene: AT4G39400
BRI1 Araport: AT4G39400
